# Ultrasensitive, Label Free, Chemiresistive Nanobiosensor Using Multiwalled Carbon Nanotubes Embedded Electrospun SU-8 Nanofibers

**DOI:** 10.3390/s16091354

**Published:** 2016-08-23

**Authors:** Matta Durga Prakash, Siva Rama Krishna Vanjari, Chandra Shekhar Sharma, Shiv Govind Singh

**Affiliations:** 1 Department of Electrical Engineering, Indian Institute of Technology Hyderabad, Hyderabad 502205, India; ee10p005@iith.ac.in (M.D.P.); sgsingh@iith.ac.in (S.G.S.); 2 Department of Chemical Engineering, Indian Institute of Technology Hyderabad, Hyderabad 502205, India; cssharma@iith.ac.in

**Keywords:** ultra sensitivity, nanobiosensors, electrospinning, MWCNTs, SU-8, chemiresistive

## Abstract

This paper reports the synthesis and fabrication of aligned electrospun nanofibers derived out of multiwalled carbon nanotubes (MWCNTs) embedded SU-8 photoresist, which are targeted towards ultrasensitive biosensor applications. The ultrasensitivity (detection in the range of fg/mL) and the specificity of these biosensors were achieved by complementing the inherent advantages of MWCNTs such as high surface to volume ratio and excellent electrical and transduction properties with the ease of surface functionalization of SU-8. The electrospinning process was optimized to precisely align nanofibers in between two electrodes of a copper microelectrode array. MWCNTs not only enhance the conductivity of SU-8 nanofibers but also act as transduction elements. In this paper, MWCNTs were embedded way beyond the percolation threshold and the optimum percentage loading of MWCNTs for maximizing the conductivity of nanofibers was figured out experimentally. As a proof of concept, the detection of myoglobin, an important biomarker for on-set of Acute Myocardial Infection (AMI) has been demonstrated by functionalizing the nanofibers with anti-myoglobin antibodies and carrying out detection using a chemiresistive method. This simple and robust device yielded a detection limit of 6 fg/mL.

## 1. Introduction

The ever growing demand for ultrasensitive label free biosensors for rapid, early diagnostic applications is being catered to by the rapid advancements in micro- and nanotechnologies that the present decade is witnessing. Development of detection methodologies which are highly sensitive, selective, simple, rapid, robust, and cost effective is the current research trend in this area [1]. Microcantilever technology has shown great promise in this regard. Several researchers have explored the platform for the detection of various biomarkers of clinical importance, both in static mode of operation as well as in dynamic mode of operation [2,3,4]. The static mode of operation relies on correlating the amount of steady state deflection of the microcantilever with the concentration of bioanalyte adsorbed onto the cantilever. In the dynamic mode of operation, detection is carried out by monitoring the change in resonance frequency of the cantilever upon the adsorption of bioanalyte. These sensors typically do not have a detection limit below 100 pg/mL. Piezoelectric excited millimeter sized cantilevers (PEMC) have been successfully utilized to achieve a sensitivity of a few femto-gram/mL (fg/mL) [5,6].

The need of specialized equipment for monitoring frequency compounded with large device dimensions may be disadvantageous in terms of bulk production and cost effectiveness. Aida et al. has proposed an impedance spectroscopy based DNA sensor with an auto-gram/mL (ag/mL) detection limit [7]. The technique is worth exploring. However, the selectivity issues of this device still need to be addressed. Optical detection methodologies involving reasonably complex instrumentation are shown to achieve low detection limits [8]. The need of the hour is to lower the detection limit using simple, robust, cost effective, point of care detection methodologies. The application of such devices are vast which include proteomic analysis, rapid early diagnosis of critical illnesses such as cancer, DNA analysis, food and environmental monitoring, etc., wherein the detection limits range are in the range of few ag/mL to fg/mL [9,10,11,12].

Nanomaterial-based biosensors have emerged as an alternative due to many unique properties that nanoscale materials offer. High surface-to-volume ratio manifests into faster kinetics. The interactions between nanomaterials and the targeted biomolecules are much more effective owing to their similar dimensions. Moreover, since the dimensions of these materials are comparable to Debye length, their surface properties affect the electronic structure significantly. Thus minute surface modification of these materials would result in change in the conductivity of the material. These properties can be exploited for designing ultrasensitive biosensors. Hence these materials are best suited for developing label free biosensors. Labelled biosensors, though sensitive, involve cumbersome, time-consuming fluorescent tagging steps and thus are not suited for point of care diagnostics. Various transduction mechanisms have been explored for developing nanobiosensors [13,14]. Among them, chemiresistive or conductometric sensors have attracted a great deal of attention because of their simple structure and their compatibility towards development of high density, multiplexed sensor arrays. In both transduction mechanisms, a noticeable change in the conductance/resistance occurs upon binding of targeted analyte molecules. However, conductometric sensors rely on the change in resistivity in ionic solution in which the target molecules are present while chemiresistive sensors rely on the change in resistance of the sensor material itself owing to the interactions.

Carbon based one-dimensional nanomaterials are potential candidates for achieving ultrasensitive biosensors. Design and development of biosensors using single walled carbon nanotubes (SWCNTs) and multiwalled carbon nanotubes (MWCNTs) have been reported [15,16,17,18,19]. Specifically for chemiresistive sensors, they are attractive because the small cross-sectional area of these nanotubes maximizes the current response along the axial direction of the nanowires resulting in large conductance changes. Garca-Aljaro et al. have demonstrated chemiresistive biosensor based on carbon nanotubes for detection of microorganisms such as E-coli and myoglobin [20,21,22]. However aligning these CNTs between conductive pads using dielectrophoresis is a time consuming and complex process. Furthermore, the number of active sites in CNTs that are available for functionalization are limited. Overcoming these aforementioned limitations could potentially result in low cost, robust, ultrasensitive biosensors since the transduction mechanism is simple and the design and fabrication technology for developing allied electronics is mature.

Conductive polymer nanowires are being explored as an alternative platform for biosensing applications. These are biocompatible, thermally stable, and have reasonably good conductivity. Furthermore, functionalization of polymers in general is a pretty straight forward process. The predominant method used for synthesizing these nanowires is electrochemical deposition process [23]. They tend to produce a bundle of nanowires rather than a single wire. Biosensors based on single wires tend to increase the sensitivity and push the limit of detection to ultra-low values. Even the minutest modification on the surface of the wire changes the physical and electronic properties of the wire drastically, thereby resulting in higher sensitivity and lower detection limits. Recently, Lee et al. have demonstrated a conductive polyaniline based nanowire sensor using a single nanowire between two conductive electrodes [24,25]. The alignment was not in situ and was carried out using electrophoresis.

Aligned nanowires derived out of mixture of CNTs and polymers combine the excellent conduction and transduction properties of CNTs with ease of functionalization and biocompatibility of polymers, thereby overcoming the limitations of both technologies. This can be achieved using electrospinning technique. Electrospinning is a simple, versatile, and widely used low cost method to produce nanofibers at large scale. A very high electric current is applied between a syringe containing polymer solution and a cathode which typically is grounded. Sub-micron fibers jet out of the syringe at a critical field when the surface tension forces are overcome by electrostatic forces. A large number of polymers have been electrospun into nanofibers [26,27,28,29,30]. The process is well established and the applications are well reviewed [31]. It is possible to align single nanofibers between two conductive posts by optimizing the electrospinning parameters [32]. The key endeavor in this work is to develop MWCNT-embedded single polymer nanofibers between two conductive electrodes. Embedding is achieved by mixing a fixed weight percentage of MWCNTs into the polymer solution. Embedding materials into electrospun fibers has been successfully demonstrated in the literature. A variety of glucose sensors were developed using electrospun nanofibers. Enzymatic glucose sensor was developed by embedding glucose oxidase (GOx) in polyvinylalcohol (PVA) nanofibers [33]. A non-enzymatic glucose sensor was demonstrated by utilizing electrospun nanofibrous membrane comprising a composite of poly(vinylidene fluoride) (PVDF) and poly(aminophenylboronic acid) (PAPBA). The cis-diol bond of boronic acid has specific affinity towards glucose. The detection of glucose was carried out using amperometry and the effect of interferences in the measurement was neglible owing to the specific interaction between PAPBA and glucose [34]. Detection of urea was carried out with electrospun nanocomposite fibers derived from urease embedded polyvinylpyrrolidone [35]. Ding et al., have reviewed comprehensively on the application of electrospun nanomaterials in sensor development [36]. Many of the polymers used in electrospinning are insulating in nature. In order to use them for transduction applications, these nanofibers should be made conductive. Several conductive nanomaterials such as MWCNTs, gold nanoparticles, graphene oxide are utilized to create nanocomposite targeted towards sensing applications. Several glucose sensors have been proposed using a variety of nanocomposites such as graphene oxide/nickel oxide nanofibers [37], PVA/chitosan/graphene oxide nanofibers [38], graphene modified PVA nanofibers [39]. Single and multiwalled carbon nanotube embedded nanofibers were synthesized for a wide range of applications spanning dielectric property improvement [40], ester hydrolysis [41], bioactive surface design [42], sensitive glucose detection [43], etc. Recent research developments in this area are thoroughly reviewed by Su et al. [44]. The focus of our work was to propose another approach to prepare sensitive biosensors. In such cases, it was imperative that the concentration of the transduction element be maximized. The transduction process was carried out with the help of MWNCT-embedded into single polymer nanofibers. The role of the polymer is to act as a coating on MWCNTs. The polymer coating can be easily functionalized, thus maximizing the amount of bioreceptors on the surface.

SU-8 (an epoxy-based negative photoresist), a well-established, well researched, commercially available polymer is chosen as the polymer for deriving the nanofibers. Apart from its regular utilization in microfabrication, SU-8 has a lot of advantages. It is biocompatible. Functionalization of SU-8 with different functional groups is well reported [45,46,47,48,49,50,51,52]. SU-8 is the most preferred material for developing flexible, sensitive, cantilever biosensors owing to its ease of patterning and functionalizing [53,54,55,56]. Several optical and acoustic biosensors are also developed using SU-8 [57,58,59]. SU-8 is available in different viscosities and can be diluted to desired viscosity using a customized thinner. SU-8 photoresist based nanofibers have been recently reported [60] and positioning of single nanofiber using electrostatic self-assembly has also been demonstrated [32]. However SU-8 inherently is insulating in nature. Embedding MWCNTs or other conductive materials would make it conductive once the concentration of embedded MWCNTs cross a percolation threshold [61,62,63].

This paper demonstrates design and fabrication of ultrasensitive nanobiosensors using MWCNT-embedded single SU-8 polymer nanofiber that is precisely aligned between two electrodes of a microelectrode array. The objective of the paper is two-fold. The first one is to figure out the maximum weight percentage of MWCNTs that can be embedded with a view to enhance initial conductivity as well as the overall sensitivity of the single nanofiber. The transduction mechanism relies on change in conductivity caused upon the binding of the analyte of interest. MWCNTs play a key role in the transduction mechanism and hence they are essential to maximizing the percentage of MWCNTs to achieve maximum sensitivity. The second objective is to demonstrate the proof of concept of ultrasensitive detection. This is achieved by functionalizing the nanofiber with antibodies of the target analyte and allowing the analyte to bind thereafter. SU-8 enhances the sensitivity by binding more target molecules onto the surface as compared to traditional sensors with MWCNTs. Thus, by complementing the inherent advantages of MWCNTs with the ease of surface functionalization of SU-8 polymer, a proof of concept for ultrasensitive detection of biomarkers of interest is demonstrated. The platform is based on immunoassay and is generic in nature. Any biomarker with a known or synthesized antibody can be detected using this platform. As a proof of concept, detection of myoglobin is demonstrated. Myoglobin, an oxygen binding globular protein typically found in skeletal and cardiac muscles, is an important marker for the confirmation of Acute Myocardial Infection (AMI). The onset of AMI increases the level of myoglobin in blood. Along with other biomarkers Creatine Kinase MB (CK-MB) and cardiac Troponin I (cTn I), this forms a set of biomarkers for early diagnosis of AMI [64].

## 2. Materials and Methods

### 2.1. Materials

Standard multi-walled carbon nanotubes (MWCNTs) with diameter range of 5–20 nm and length 1–10 µm was purchased from Reinste Nano Ventures Pvt. Ltd. (New Delhi, India). An epoxy-based negative photoresist, SU-8 series formulation (cyclopentanone based), was obtained from Micro Chem, Newton, MA, USA. SU-8 is available in several viscosities, SU-2002 being the lowest and SU-2150 being the highest. For our experimentation SU-8 2015 is procured as it suits our viscosity requirements. S1813 photoresist was also procured from Micro Chem, Newton, MA, USA. Phosphate buffered saline tablets (PBS), 1-Ethyl-3-(3-dimethylaminopropyl) carbodiimide (EDC), *N*-Hydroxysuccinimide (NHS), chloroform, Bovine Serum Albumin (BSA), Myoglobin from equine skeletal muscle and monoclonal antibody myoglobin purchased from Sigma Aldrich, India. Ultra pure DI water (18.2 MΩ·cm) was used throughout.

### 2.2. Fabrication of Microelectrode Array

**Figure 1:**
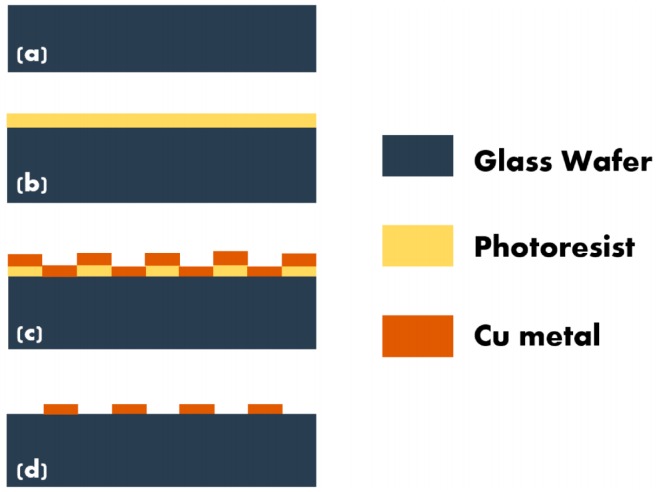
Schematic representation of process flow for the fabrication of Microelectrode Array: (**a**) Glass substrate; (**b**) Sacrificial layer; (**c**) Patterning the sacrificial layer and (**d**) deposition of the target material.

In order to perform electrical characterization of nanofibers, it is essential to precisely position the fiber between two conductive regions. For this purpose, copper microelectrode arrays were fabricated onto a glass substrate using standard lift off technique. The schematic representation of the same is depicted in Figure 1. Initially the glass substrates were cleaned sequentially in acetone and IPA for 5 min each followed by drying with ultra-high pure nitrogen and dehydration bake at 100 °C for 10 min. Positive photoresist S-1813 was spin coated on it at 500 rpm 10 s, and 3000 rpm, 35 s. This was followed by a prebake at 115 °C 60 s, UV exposure using the mask having desired pattern and development. The resultant patterned substrate was loaded into sputtering system immediately and 50 nm Ti/200 nm Cu were deposited sequentially. The last step in the process was immersion of this substrate in acetone and ultrasonication of the same, which resulted in lifting off Ti/Cu in unwanted regions. The diameter of the electrodes in the fabricated microelectrode array was 200 µm and the spacing between two electrodes was 50 µm. Since the nanofibers emanating from the polymer jet are charged, they tend to terminate onto conductive surfaces rather than insulating surfaces. Researchers have demonstrated precise alignment of single nanofibers between conductive posts fabricated out of RF gel by optimizing the electrospinning parameters [30]. In this work, conductive microelectrodes were fabricated using copper which is more conductive and easy to fabricate.

### 2.3. Synthesis and Alignment of Nanofibers

The next task was to synthesize MWNCT-embedded SU-8 nanofibers using electrospinning (E-spin Nanotech Pvt. Ltd., Kanpur, India) and to carry out in-situ alignment of single nanofiber between two electrodes of the microelectrode array. For this purpose, the glass substrate patterned with Cu microelectrode array was used as the collector. The polymer solution that was loaded into the syringe of the electrospinning set up is a mixture of SU-8 and MWCNTs. This mixture was prepared by initially dispersing a known weight percentage of MWCNTs in chloroform and then sonicating the mixture of the prepared dispersion and SU-8 2015 using a probe sonicator. The role of the solvent was to aid dispersion on MWCNTs in SU-8 and probe sonication ensured proper dispersion of MWCNTs into SU-8. By tuning the electrospinning parameters such as the collector voltage, the tip to collector distance, the deposition time, and the flow rate of the solution, single nanofibers were precisely aligned between two electrodes. With a view to maximize the conductivity of the nanofiber, the weight percentage of MWCNTs in the polymer solution was increased up to an extent till electrospinning of controlled nanofiber morphology was possible. The maximum weight percentage that could be mixed was 13%, beyond which the syringe was getting completely clogged. Two probe method was adapted for electrical characterization and was carried out using Keithley 4200 semiconductor characterization system. Two probe method involves application of current/voltage to the two conductive pads using micromanipulators and measurement of the resultant voltage/current across the pads using the same micromanipulators. Both the stimulus and recording were carried out using Keithley 4200 semiconductor characterization system which was connected to the micromanipulators. The conductance/conductivity was measured by taking the ratio of applied/measured current and the measured/applied voltage.

### 2.4. Surface Functionalization

The functionalization of the aligned nanofiber for immobilizing of monoclonal antibodies (mAbs) of myoglobin was carried out using well established EDC, NHS chemistry [23,45]. Prior to functionalization, several single MWCNT-embedded SU-8 nanofibers were soaked in HCl of 0.1 M for 10 min to increase the surface reactivity. A mixture solution of EDC/NHS (0.2/0.2 M) with the mAbs of myoglobin was dropped on top of the aligned nanofibers and was kept for 6 h at room temperature in a dark area. Then the MWCNT-embedded SU-8 nanofibers were washed using a deionized water to eliminate non-immobilized mAbs on the nanofibers, Cu electrodes and glass substrates which completed the immobilization process. The concentration of mAbs was chosen as 200 µg/mL for these experiments. All the non-specific binding sites were blocked with Bovine serum albumin (BSA).

### 2.5. Experimentation Methodology

**Figure 2:**
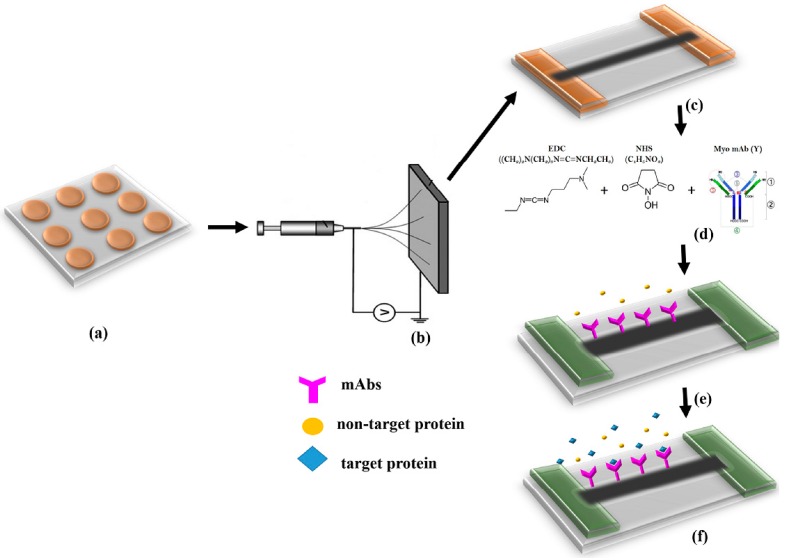
Schematic illustration of functionalized MWNCT-embedded SU-8 nanofibers for detection of myoglobin. (**a**) Microelectrode Array; (**b**) Electrospinning set-up; (**c**) Aligned nanofiber between electrodes; (**d**) EDC, NHS Chemistry for Antibody Immobilization; (**e**) Myoglobin Antibody immobilized onto nanofiber; (**f**) Binding of Myoglobin onto the nanofiber.

Figure 2 depicts the entire process flow involving the device fabrication protocol, the functionalization protocol and detection methodology. MWNCT-embedded SU-8 electrospun nanofibers were aligned between two electrodes of a copper microelectrode array fabricated on a glass substrate. The parameters of electrospinning were optimized to ensure a single nanofiber between two electrodes. The nanofiber was characterized thoroughly using several physiochemical and electrical characterization techniques. The key focus of electrical characterization was to figure out the maximum amount of MWCNTs that can embedded inside the polymer. For this purpose, nanofibers containing various weight percentages of MWCNTs have been synthesized and characterized. Subsequent to characterization, the functionalization of nanofiber with monoclonal antibodies (mAbs) of myoglobin was carried out using standard EDC and NHS protocol [23]. The chemiresistive/conductometric detection of myoglobin was carried out using standard two probe measurements. Each of these experiments were conducted at least five times to ensure repeatability.

## 3. Results and Discussion

### 3.1. SEM Analysis

**Figure 3:**
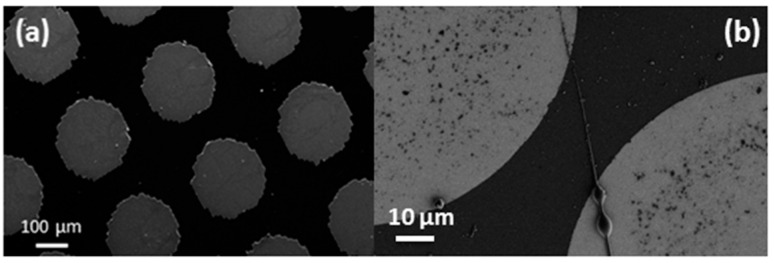
SEM images: (**a**) Cu microelectrodes array; (**b**) Aligned nanofiber between two electrodes.

Prior to carrying out conductivity measurements, the surface morphology of these synthesized fibers was analyzed using SEM (Quanta 200, FEI, Frankfurt, Germany). Figure 3a shows the SEM image of microfabricated Cu microelectrode array. Figure 3b shows a single nanofiber that is precisely aligned between two Cu electrode arrays. The weight percentage of MWCNTs in this case is 8%. This was achieved by optimizing the electrospinning parameters. The diameter of MWNCT-embedded SU-8 nanofiber was narrowly distributed with mean diameter of 280 ± 28 nm.

### 3.2. HRTEM Analysis

**Figure 4:**
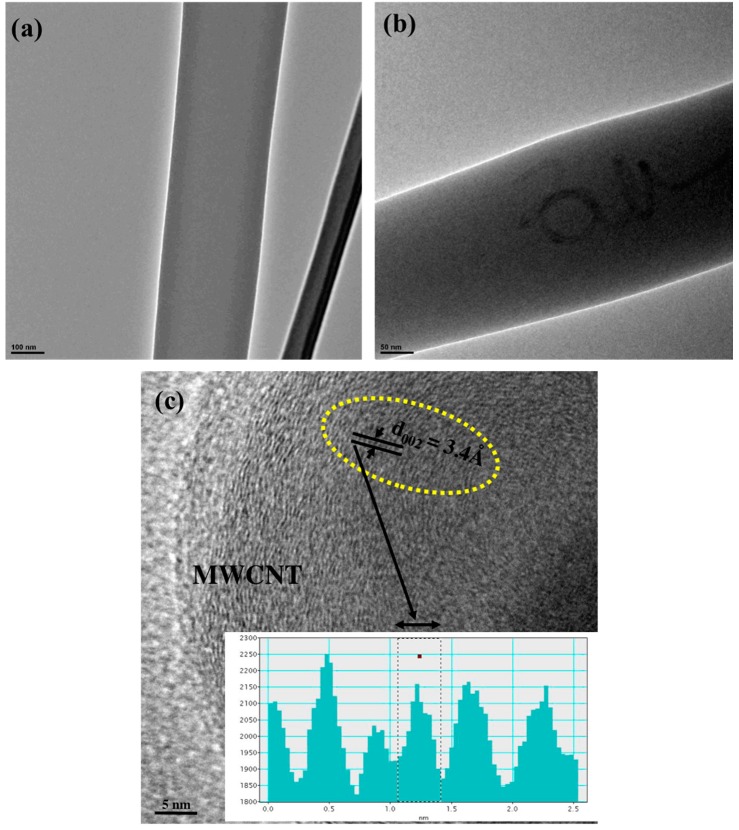
HRTEM analysis of: (**a**) SU-8 electrospun nanofiber; (**b**) MWNCT-embedded electrospun SU-8 nanofiber; (**c**) An atomic-scale of a MWCNT depicting interlayer spacing of 0.34 nm.

To understand the internal structure of the MWCNT/SU-8 composite nanofibers, TEM analysis (Philips, CM200, Eindhoven, The Netherlands) was carried out on the nanofiber samples extracted for this purpose. The fibers were directly spun onto TEM lacey grids. Figure 4 shows the TEM images of pure SU-8 fibers and multiwalled carbon nanotube embedded SU-8 fibers. In Figure 4b a random path formed by MWCNTs inside the polymer matrix can be observed which is absent in Figure 4a. Figure 4c shows the high-resolution image of the individual MWCNTs shows inter layer spacing of 0.34 nm. All these images clearly reveal the successful formation of the MWNCT-embedded SU-8 nanofibers.

### 3.3. XRD Analysis

**Figure 5:**
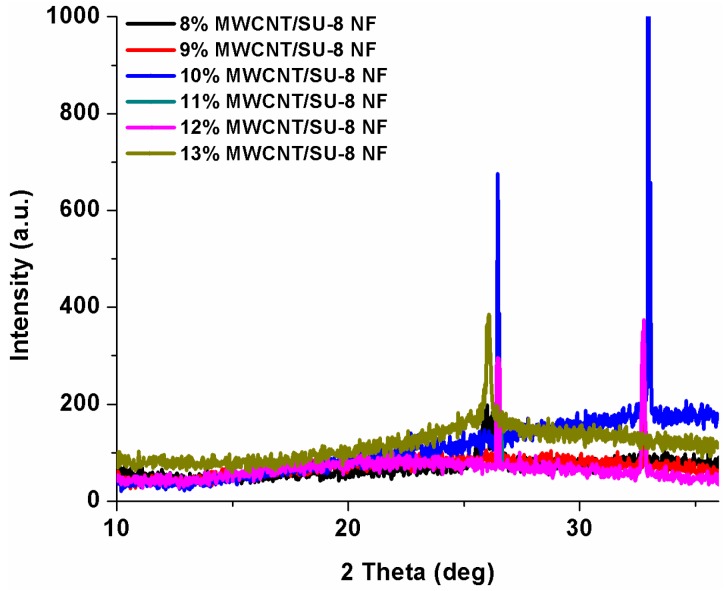
X-ray Diffraction Analysis of MWCNTs/SU-8 nanofibers.

The morphology of embedded MWCNTs was also characterized using XRD apart from SEM and TEM analysis. For XRD analysis Nanofibers having various weight percentage of MWCNTs were spun on a blank p-type silicon substrate. X-ray diffraction (XRD) analysis of powders was carried out using Xpert Pro X-ray diffractometer with monochromatic CuK_α_ radiation. Figure 5 shows the XRD data for as spun nanofibers for different weight percentages of MWCNTs. The presence of peak at 2θ = 26° at all weight percentages of CNTs is a reaffirmation of the presence of MWCNTs in their original crystalline form with a (0, 0, 2) orientation. MWCNTs were not damaged because of the application of a high electric field and they were not agglomerated also.

### 3.4. Conductivity Enhancement of SU-8 Using MWCNTs

**Figure 6:**
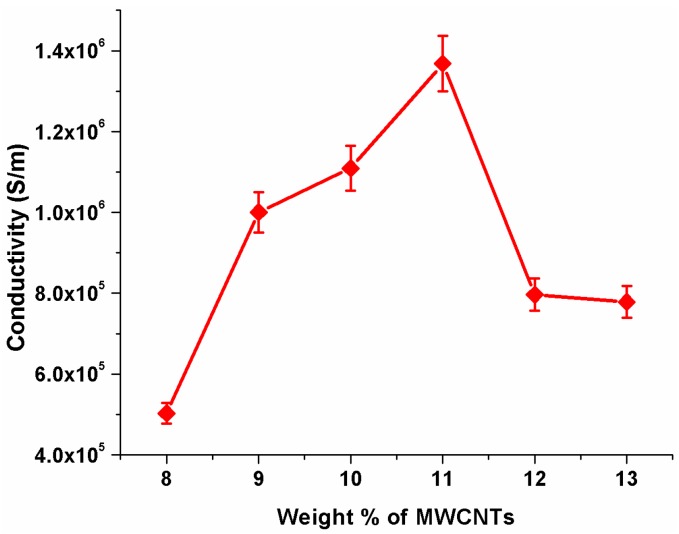
Variation of Electrical Conductivity as a function of MWCNTs weight.

With an emphasis on maximizing the conductivity of MWNCT-embedded SU-8 nanofiber, the weight percentage of MWCNTs in the polymer solution prior to spinning was gradually increased and conductivity of single aligned electrospun nanofiber was measured using a simple two probe method. Figure 6 depicts conductivity of the nanofiber as a function of weight percentage of MWCNTs.

**Figure 7:**
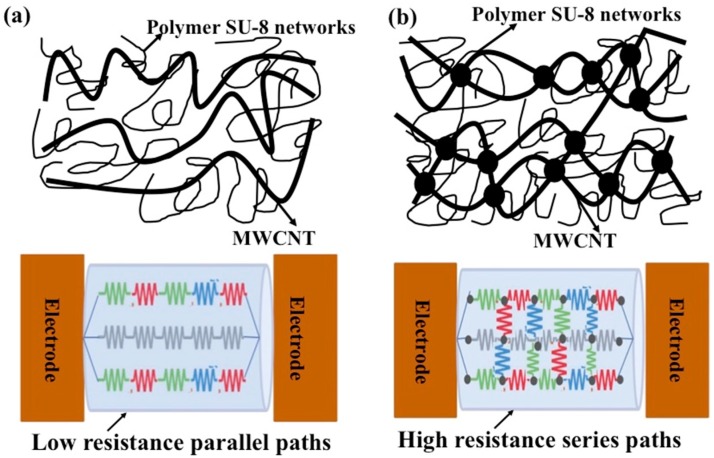
A resistor network model for MWNCT-embedded SU-8 nanofiber co-percolating system: (**a**) Low resistance parallel paths; (**b**) Interconnection of parallel paths leading to high resistance percentage.

Interestingly, the conductivity behavior was found to be nonlinear with respect to the concentration of MWCNTs. It was maximum at 11% weight percentage and reduced beyond that. The conductive nature of nanofibers at 8% MWCNTs indicates that the concentration of MWCNTs is beyond the percolation threshold. The conductive paths in a percolating can be described with a network of resistors [43]. Since the MWCNTs were embedded randomly, there might be several parallel paths that allowed the flow of charges at relatively low weight percentages of MWCNTs as shown in Figure 7a. In the schematic, the smaller, thinner strands represent the polymer matrix inside the nanofiber and the thicker lines represent MWCNTs that are embedded inside the nanofiber. The resistivity of each path might be different owing the random ordering of MWCNTs. However, since these paths are in parallel, the resistivity and hence conductivity is governed by the least resistive path. Beyond a certain weight percentage, the concentration of MWCNTs is so high that there is a possibility of MWCNTs connecting these parallel paths as shown in Figure 7b. In such a case the conductivity is not governed by the least resistive path. The highly resistive path would also contribute, thus leading to decrease in the conductivity. Furthermore, at higher weight percentages, mobility degradation of charge carriers due to lattice scattering is a well-known phenomenon that degrades the conductivity. For subsequent experimentation, the concentration of MWCNTs was fixed at 11% *w*/*w* as it yielded the maximum conductivity.

### 3.5. Detection of Myoglobin

**Figure 8:**
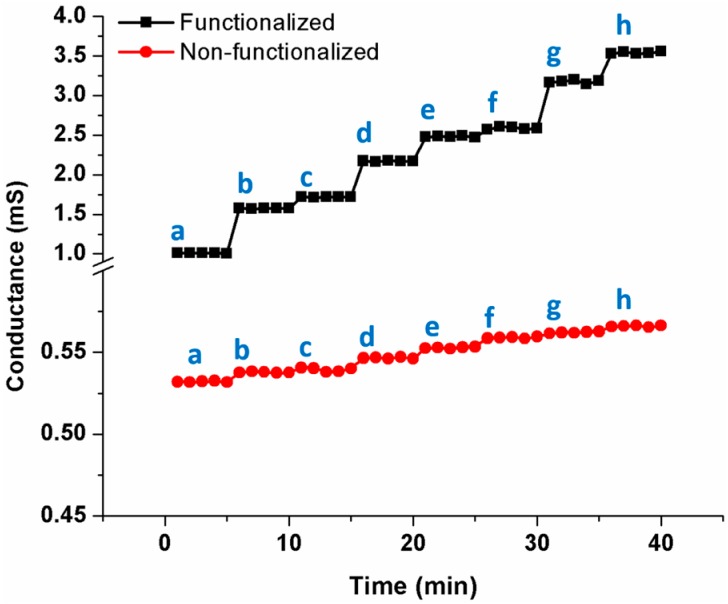
Detection of myoglobin on single functionalized MWCNT/SU-8 nanofiber (a: 6 fg/mL; b: 10 fg/mL; c: 100 fg/mL; d: 10 pg/mL; e: 100 pg/mL; f: 10 ng/mL; g: 100 ng/mL; and h: 10 ug/mL); Variation in the case of non-functionalized nanofiber is random.

In order to test the efficacy of the device for biosensing applications, the aligned nanofiber was functionalized with the antibodies of myoglobin using the protocol described in the experimental section. The phosphate buffer saline (PBS) buffer of pH 7.0 was used as a supporting electrolyte. The sensing methodology adapted can be categorized either as a chemiresistive or conductometric, the former being more appropriate and was proven with the following results. In both the cases, the transduction mechanism was the change in conductivity upon the binding of analyte of interest. While the chemresistive method relies on the change in the conductivity of the nanofiber itself, the conductometric method on the other hand relies on the change in the conductivity of the medium due to the addition of the analyte. Initially, the resistance of functionalized, aligned, single nanofiber was measured in pure PBS buffer solution. The resistance of nanowire in the buffer solution was measured to be 993.8 Ω. Upon the sequential addition of myoglobin, the resistance decreased, thus improving the conductance as shown in Figure 8. Upon the binding of myoglobin, the surface stress on the nanofiber increases which in turn increases the mobility of charge carriers in MWCNTs, thus increasing the conductivity. In Figure 8, two graphs at different scales are represented in the same graph for the sake of clarity. There was a minimal variation of conductance when the entire experiment was repeated on a non-functionalized nanofiber.

**Figure 9:**
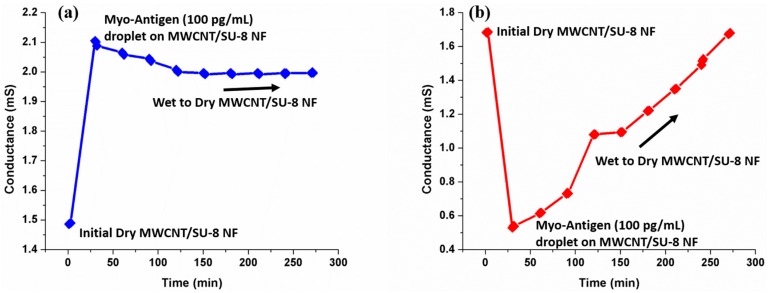
Variation of conductance with respect to time for a concentration of 100 pg/mL myglobin on (**a**) Functionalized nanofiber (**b**) Non-functionalized Nanofiber.

There is a possibility that the change in conductance in the functionalized as well as non-functionalized wire is due to change in the conductivity of the solution owing to the addition of myoglobin which is a charged globular metalloproteine. To reaffirm the selectivity, firstly the conductance of nanofiber without supporting electrolyte was measured. After that a fixed concentration of myoglobin was added along with the supporting electrolyte and allowed to evaporate with time. Conductance was measured at regular intervals till the electrolyte was completely evaporated and the nanofiber is dry. As observed from Figure 9a, the conductance of the nanofiber did not come back to its original dry state condition value for functionalized nanofiber and for non-functionalized nanofiber, the conductance restored to its original value. Two inferences can be made out of these experiments. The selectivity of the biosensor is established as the conductance was altered upon the addition of myoglobin onto an anti-myoglobin functionalized nanofiber and it remained unaltered in the case of non-functionalized nanofiber as shown in Figure 9b. This confirms the binding of myoglobin onto the surface of the nanofiber. Since the platform was based on immunoassay technique, we did not carry out the specificity test with other antigens as immunoassay protocols were known to be highly specific. The second inference is that the mechanism of transduction can be categorized as chemiresistive as the surface conductivity is affected rather than the bulk conductivity. The experiments were repeated several times and the CV in the conductivity measurements was well within 5%.

**Figure 10:**
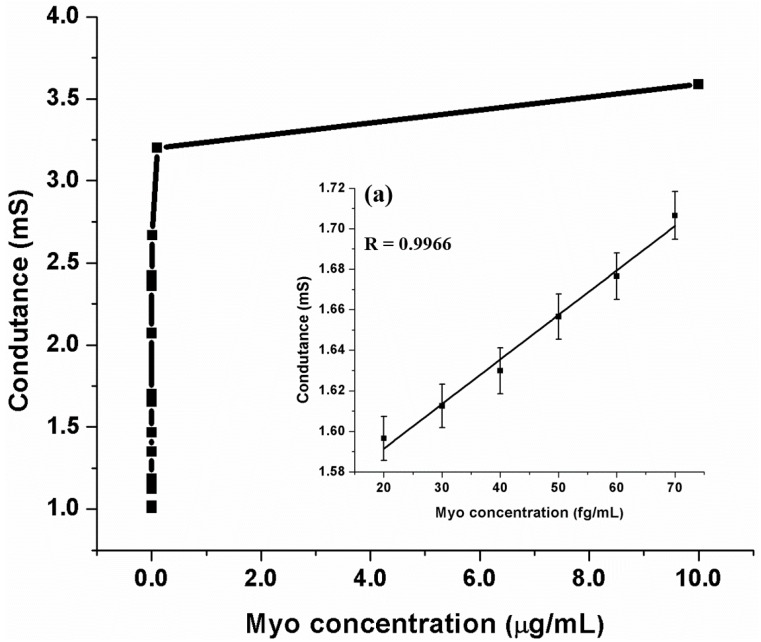
Myoglobin in broad detection range performance. (**a**) Linearity range of Nanobiosensor for myoglobin: 20–70 fg/mL.

The proposed biosensor achieved a detection limit of 6 fg/mL and had a range of detection from fg/mL to µg/mL as shown in Figure 10. The limit of detection was calculated using standard sigmoidal binding curve analysis [65]. In order to study the stability of our platform, the synthesized and functionalized wires were stored under 4° for more than a month and the experiments were repeated. The results are well within the error bars of the previous measurements.

Table 1 shows the comparison of the proposed methodology with the several sensing methodologies for myoglobin in the existing literature [66,67,68,69,70,71]. Through our simple platform, we are able to demonstrate an ultrasensitive detection of myoglobin with a limit of detection that is far superior to any of the existing methodologies. As mentioned earlier, proof of concept is demonstrated using Myoglobin. The linearity range was obtained in the range of 20 fg/mL to 70 fg/mL with a correlation coefficient of 0.9953. The linearity range may be further improved by carrying out optimization of antibody binding protocol. This generic immunoassay platform can be utilized for any other biomarker of interest where ultrasensitivity is paramount.

## 4. Conclusions

In conclusion, synthesis and fabrication of aligned nanofibers derived out of MWNCT-embedded SU-8 photoresist was carried out and its application towards ultrasensitive chemiresistive biosensor was demonstrated. The ultra-sensitivity (detection of few fg/mL) and the specificity of these biosensors was a result of exploiting the advantages of transduction properties of MWCNTs and the ease of surface functionalization of SU-8 polymer simultaneously. The device fabrication is simple and robust as it involves fabrication of a microelectrode array and an electrospinning technique which are well established processes. The parameters of electrospinning were optimized to precisely align nanofibers in between two electrodes of a copper microelectrode array fabricated on a glass substrate. MWCNTs were embedded in a polymer nanofibers way beyond the percolation limit. However, the conductivity tends to decrease beyond a certain weight percentage as demonstrated in the case of MWNCT-embedded SU-8 nanofibers. This is because of the mobility degradation caused by the random orientation of MWCNTs in the SU-8 polymer matrix. As a proof of concept, the detection of myoglobin, an important biomarker for various diseases, TEM lacaey grids was demonstrated using chemiresistive detection methodology with a detection limit of 6 fg/mL.

## Figures and Tables

**Table 1:** Myoglobin detection on different transduction platforms and their detection range reported in the literature and the present reported work.

Transduction Platform	Detection Range	Ref.
ELISA	20–230 ng·mL^−1^	[66]
	LOD—16 ng·mL^−1^	
Chemiluminescence	10–10^4^ ng·mL^−1^	[67]
	LOD—1.2 ng·mL^−1^	
Fluorescence	0.01–10 ng·mL^−1^	[68]
	LOD—0.01 ng·mL^−1^	
Surface plasmon resonance (SPR)	0.1–200 ng·mL^−1^	[69]
	LOD—below 1 ng·mL^−1^	
Electrochemical Impedance Spectroscopy/interdigitated electrodes	0.5–500 ng·mL^−1^	[70]
	LOD—100 ng·mL^−1^	
Electrochemical/nanoparticles modified electrodes	17.8–1780 ng·mL^−1^	[71]
	LOD—5 ng·mL^−1^	
**Chemiresistive** **(Present work)**	**20–70 fg·mL^−1^**	
	**LOD—6 fg·mL^−1^**	
